# COVID-19 Vaccine Knowledge and Attitude Among Healthcare Workers in Jeddah, Saudi Arabia

**DOI:** 10.7759/cureus.41070

**Published:** 2023-06-28

**Authors:** Abdulhakeem Althaqafi, Adeeb Munshi, Mohamed K Mujalled, Enas Munshi, Ahmad Alhouthali, Lama Alqalayta, Hala Zahed, Mariya S Bahashwan, Laila Alghubayshi, Ahmad Alghamdi

**Affiliations:** 1 Medicine, King Saud Bin Abdulaziz University for Health Sciences, Jeddah, SAU; 2 Infectious Diseases, King Abdullah International Medical Research Center, Jeddah, SAU; 3 Medicine/Infectious Diseases, King Abdulaziz Medical City, Jeddah, SAU; 4 Internal Medicine, King Abdulaziz Medical City, Jeddah, SAU; 5 Infection Prevention and Control, King Abdullah International Medical Research Center, Jeddah, SAU; 6 Infection Prevention and Control, King Abdulaziz Medical City, Jeddah, SAU

**Keywords:** healthcare worker (hcw), saudi arabia, attitude, knowledge, vaccination, covid-19

## Abstract

Background

On March 11, 2020, the World Health Organization (WHO) proclaimed coronavirus disease 2019 (COVID-19) a pandemic. COVID-19 can result in asymptomatic infection, severe pneumonia, and death. In this study, healthcare workers in Jeddah, Saudi Arabia, were asked to reflect on their knowledge of and attitudes toward the COVID-19 vaccine.

Methods

Online anonymous polling of healthcare professionals in Jeddah, Saudi Arabia, was done. During the COVID-19 pandemic, the respondents' demographic information and knowledge of and attitudes toward the COVID-19 vaccine were gathered through a questionnaire. Both descriptive and inferential statistics were used to analyze the data.

Results

The knowledge of the responders for each vaccine is as follows: Pfizer-BioNTech, 96.1%; AstraZeneca-Oxford, 66.1%; Moderna, 56.9%; and Johnson & Johnson's Janssen, 18.2%. A small percentage of responders (5.7%) believe that COVID-19 vaccination may alter their DNA, while 70% believe it can prevent them from getting sick with COVID-19 infection. Half of the responders had a wrong opinion of COVID-19 vaccinations.

Conclusion

The findings imply that improving healthcare workers' knowledge of vaccines and changing attitudes toward vaccination may increase vaccine acceptability. This would involve addressing the respondents' concerns about vaccine side effects and their general mistrust of vaccine benefits.

## Introduction

The World Health Organization (WHO) declared coronavirus disease 2019 (COVID-19) a pandemic on March 11, 2020 [1]. COVID-19 can cause asymptomatic infection, severe pneumonia, and death [2]. In March 2020, the Kingdom of Saudi Arabia (KSA) reported its first COVID-19 case [3].

The nation saw three waves: the first wave began in March 2020, the second wave started in January 2021, and finally, the third (Omicron) wave began in January 2022 [4].

A long-awaited vaccine against COVID-19 has become available, and many countries, including the Kingdom of Saudi Arabia (KSA), have already reserved it. Pfizer-BioNTech's COVID-19 vaccine has been approved by the Saudi Food and Drug Authority, and the country has deployed phased vaccinations. The vaccine was administered early to healthcare workers, older people, and those with chronic and autoimmune disorders [5]. The success of any immunization plan depends on the acceptance and uptake of an emergency-released vaccine. Without this assurance, vaccine reluctance is inevitable [6].

Healthcare professionals are crucial to the effectiveness of immunization programs, and research has shown that their attitudes toward and knowledge of vaccinations influence both their intentions to offer the vaccine and their choices to acquire it [7,8]. Numerous studies have shown that healthcare professionals can be vaccine-apprehensive themselves and that their reluctance might influence public hesitancy and opposition to vaccination [9-11]. Additionally, it has been noted that healthcare professionals who have unfavorable opinions regarding vaccinations and are opposed to or are hesitant about recommending them to patients tend to do so infrequently [12].

Several COVID-19 vaccines, including those made by AstraZeneca, Pfizer-BioNTech, Moderna, and Johnson & Johnson (Janssen), have already been developed in several nations. Several nations have received these vaccinations. The success of immunization programs in achieving high vaccination coverage rates, particularly for recently emerging infectious diseases, depends on the perception of disease risk and vaccine demand in populations [13-15]. As vaccine refusal would expose more pupils to the disease, such information aids appropriate authorities in making educated forecasts regarding vaccine acceptance and developing methods for enhancing acceptability.

Refusal to receive vaccinations is a problem that affects the entire world [16,17]. To overcome vaccine hesitancy and foster confidence in vaccination, the World Health Organization suggests taking a proactive approach [18,19]. Understanding the causes of resistance to the use of COVID-19 vaccine and how to overcome this resistance is necessary to control or eliminate the pandemic through a vaccination program.

Knowing differing vaccine attitudes and information is essential because a heterogeneous approach to vaccine refusal that addresses the concerns of various groups is more successful than a homogeneous one [20,21]. Furthermore, given Saudi's recent vaccine deployment, it is vital to have a qualitative and quantitative assessment of vaccine knowledge. Therefore, the current study examined the relationship between knowledge of, attitude toward, and acceptance of the COVID-19 vaccine among healthcare workers in Jeddah, Saudi Arabia.

## Materials and methods

Study design

A descriptive survey design was adopted for this study. Using this method, researchers may explain the traits of a population or the variations among Jeddah's healthcare professionals, and they can also anticipate the future using the correlation survey data. In addition, the reliability of this design in preserving the respondents' anonymity, which motivates them to provide truthful replies, made it appropriate for this study.

Study population

The questionnaire was distributed among healthcare workers in Jeddah. All workers in hospitals were eligible for participation in the study, including physicians, nurses, respiratory therapists, and paramedics.

Informed consent was obtained from all participants for inclusion in the study.

The participants were clearly informed about the study's purpose and background on the online questionnaire's first page. In the questionnaire, the respondents were informed that they could withdraw at any time without providing a reason and that all information and opinions provided would remain anonymous.

Instrument

The study used a structured questionnaire titled "Knowledge and Attitude of COVID-19 Vaccine Among Healthcare Workers in Jeddah." Furthermore, several instrument components were self-developed, even if some questionnaire items were modified to reflect their application in other countries and circumstances. For example, the attitude scale was adapted from Paul et al. [22] with Cronbach's alpha values of 0.91-0.94, while the knowledge scale was adapted from the Centers for Disease Control and Prevention [23]. The questionnaire consisted of three parts. The first part is about demographic information such as sex, age, profession, and vaccination status against COVID-19. The second part included six questions, which assessed the responder's knowledge of the COVID-19 vaccine. The third part consisted of four questions, and it aimed to assess the healthcare worker's attitudes toward the COVID-19 vaccine. The questionnaire was conducted in English. For validity, the questionnaire was created by an infectious disease expert and reviewed by two experts in infection control and public health.

Data collection

A standardized, well-structured, self-administered questionnaire was used to provide data using Google Forms (Google, Inc., Mountain View, CA). Given that WhatsApp (Meta Platforms, Inc., Menlo Park, CA) is the professors' primary social media medium to connect with healthcare professionals in Jeddah, the survey link was distributed through this platform.

Statistical analysis

Data were simultaneously entered into a preform and updated. It was entered into Microsoft Excel (MS Office 2010) (Microsoft® Corp., Redmond, WA). The data were analyzed using Statistical Package for Social Sciences (SPSS) software version 22.0 (IBM SPSS Statistics, Armonk, NY). Descriptive analysis included the computation of frequencies and percentages. The number was used to represent categorical variables, whereas quantitative variables were described using mean ± standard deviation (SD).

Ethical consideration

The study was approved by the Institutional Review Board (IRB) of King Abdullah International Medical Research Center (KAIMRC). The study number is NRJ22J/011/01.

## Results

The questionnaire was circulated among healthcare workers in Jeddah. A total of 230 responses were collected and subsequently used in the analysis.

The findings in Table 1 show that the majority of the respondents were 69.6% male and 30.4% female. Most respondents were, on average, from 25 to 30 years (168/320, 73.0%), and 26 respondents were, on average, from 31 to 35 years. Two hundred six respondents (89.6%) received three doses of vaccine against COVID-19. The demographic data are shown in Table 1.

**Table 1:** Demographic information of the respondents COVID-19: coronavirus disease 2019

Variable	Number	Percentage (%)
Sex		
Male	160	69.6
Female	70	30.4
Total	230	100
Age		
25-30	168	73.0
31-35	26	11.3
36-40	8	3.5
41-45	8	3.5
More than 46	20	8.7
Total	230	100
Vaccinated against COVID-19		
Yes, one dose	1	0.4
Yes, two doses	21	9.1
Yes, three doses	206	89.6
No	2	0.9
Total	230	100
Profession		
Physician	198	86
Nurse	20	8.7
Respiratory therapist	6	2.6
Paramedics	6	2.6

Table 2 shows general knowledge about the COVID-19 vaccine; the percentage of the responders who know each vaccine was as follows: Pfizer-BioNTech, 96.1%; AstraZeneca-Oxford, 66.1%; Moderna, 56.9%; and Johnson & Johnson's Janssen, 18.2%. Of the responders, 5.7% answered yes when they were asked if COVID-19 vaccination may alter their DNA, 70% of the responders believe that the vaccination can prevent them from getting sick with COVID-19 infection, 10% of the responders believed that they do not need to get vaccinated if they already had COVID-19 infection, 75.2% of the responders believe that they could still test positive for COVID-19 on a viral test after receiving the vaccine, and 17% of the responders believe that they can get sick from the vaccine itself.

**Table 2:** Knowledge of COVID-19 vaccination COVID-19: coronavirus disease 2019

Serial number	Knowledge of the COVID-19 vaccine	Yes	No
1	Approved COVID-19 vaccines		
	AstraZeneca-Oxford COVID-19 vaccine	152 (66.1%)	78 (43.9%)
	Pfizer-BioNTech COVID-19 vaccine	221 (96.1%)	9 (3.9%)
	Johnson & Johnson's Janssen COVID-19 vaccine	42 (18.2%)	188 (81.8%)
	Moderna COVID-19 vaccine	131 (56.9%)	99 (43.1%)
2	COVID-19 vaccine can alter my DNA	13 (5.7%)	217 (94.3%)
3	COVID-19 vaccination can protect me from getting sick with COVID-19	161 (70.0%)	69 (30%)
4	I do not need to get vaccinated if I already have COVID-19	23 (10.0%)	207 (90.0%)
5	After getting a COVID-19 vaccine, I can still test positive for COVID-19 on a viral test	173 (75.2%)	57 (24.8%)
6	COVID-19 vaccine can make me sick with COVID-19	39 (17.0%)	191 (83.0%)

The results from Table 3 show that the majority of the respondents have attitudes toward the COVID-19 vaccine that include concerns about unintended consequences (mean = 2.5), a preference for natural immunity (mean = 2.5), a general mistrust of the benefits of vaccination (mean = 2.9), and worries about commercial profiteering (mean = 2.4). The majority of the responders had a wrong opinion about COVID-19 vaccinations.

**Table 3:** Attitudes toward COVID-19 vaccine A, agree; D, disagree; M, mean; SD, strongly disagree; SA, strongly agree; SD, standard deviation; COVID-19, coronavirus disease 2019

Serial number	Concern	SA	A	D	SD	M	SD
1	General mistrust of vaccine benefit	15 (6.5%)	63 (27.4%)	77 (33.5%)	75 (32.6%)	2.9	0.9
2	Worries about unforeseen effect	22 (9.6%)	97 (42.2%)	81 (35.2%)	30 (13.0%)	2.5	0.8
3	Concern about commercial profiteering	27 (11.7%)	102 (44.3%)	68 (29.6%)	33 (14.3%)	2.4	0.8
4	Preference for natural immunity	29 (12.6%)	83 (36.1%)	83 (36.1%)	35 (15.2%)	2.5	0.9

## Discussion

Healthcare professionals are essential to the success of vaccination programs, and research has shown that their attitudes toward vaccination and knowledge of them have an impact on both their intentions to provide the vaccine and their decisions to do so. Numerous studies have demonstrated that medical professionals may also have vaccine hesitancy and that this hesitation may have an impact on public hesitancy and opposition to vaccination [9-11]. Furthermore, it has been noticed that medical practitioners who are opposed to, disagree with, or refuse to prescribe vaccination to patients tend to do so infrequently [12].

In the present study, 168/320 respondents, or 73.0%, were, on average, between 25 and 30 years old, and 26 respondents were between 31 and 35 years. The majority of the respondents were 69.6% male and 30.4% female. These results agreed with Qattan et al. [24], who reported that most of the healthcare worker participants were aged 30-49 (45.32%) and were male (60.18%).

Our study showed that the Pfizer-BioNTech COVID-19 vaccine was the most known vaccine among the participants (96.1%), followed by AstraZeneca-Oxford (66.1%), Moderna (56.9%), and Johnson & Johnson's Janssen (18.2%). Saddik et al. reported that the knowledge level among healthcare workers in the United Arab Emirates (UAE) about the COVID-19 vaccine was 79% [25]. Another study in Saudi Arabia found that there is insufficient understanding of the COVID-19 vaccine among healthcare workers. The AstraZeneca-Oxford COVID-19 vaccine was known to 40% of the Saudi healthcare workers, and the Pfizer-BioNTech COVID-19 vaccine was known to only one-third [26]. Our study showed significant knowledge of the COVID-19 vaccine, especially Pfizer-BioNTech and AstraZeneca-Oxford. This is because these two vaccines were first approved in Saudi Arabia.

Of the healthcare workers in our study, 6.5% had general mistrust of the COVID-19 vaccine benefit, while 9.6% worried about unforeseen side effects. This is in contrast to a survey conducted in the UAE, where a significant proportion of the population reported concerns about the safety, side effects, and ineffectiveness of the COVID-19 vaccine [25]. Additionally, many worries and doubts about safety and adverse effects were reported from Australia and Europe [6,27].

The survey results confirmed our prediction that specific vaccine information would influence vaccination intention. These results were in contrast to those of Pogue et al. [28], who found no significant correlation between knowledge score and choice to vaccinate. The study backs up the findings of Gallè et al. [29], who discovered a tangible link between vaccine acceptance and knowledge.

As healthcare workers' knowledge of and attitudes toward vaccines are crucial to their likelihood of advocating vaccination to their patients, this study and other similar studies highlight the need for healthcare workers to be more educated and confident about vaccines.

The limitations of our investigation exist. One limitation is that an online survey could limit the representativeness of the current healthcare worker's sample, since an offline survey was not practical under the social distancing mandate. To solve this issue, we enlisted a sizable sample size and applied a straightforward random sampling strategy throughout Jeddah to increase sample diversity and representativeness. Another limitation is that because the study is speculative, the results could differ from experience, and specific self-reported replies might introduce bias into the data.

The convenience sampling technique was a considerable a limitation of our study because it limits the generalizability of the data, making it very difficult to represent all healthcare workers in Jeddah, Saudi Arabia.

Finally, a cross-sectional design, which limits the identification of the causal effect on outcomes, and the small sample size were additional study limitations. So, prospective and longitudinal studies are recommended in this regard.

## Conclusions

The study concludes that vaccination rates and healthcare workers' knowledge of COVID-19 vaccinations are favorably correlated. A variety of licensed vaccines were found to be familiar to healthcare professionals. In addition, healthcare workers had a higher intention to immunize and were more likely to have done so. The COVID-19 vaccine is an ideal way to end the COVID-19 infection. So, encourage healthy workers to get vaccinated to lower their risk of contracting COVID-19.
